# Pathobiology of Hodgkin Lymphoma

**DOI:** 10.1155/2011/920898

**Published:** 2010-12-22

**Authors:** Pier Paolo Piccaluga, Claudio Agostinelli, Anna Gazzola, Claudio Tripodo, Francesco Bacci, Elena Sabattini, Maria Teresa Sista, Claudia Mannu, Maria Rosaria Sapienza, Maura Rossi, Maria Antonella Laginestra, Carlo A. Sagramoso-Sacchetti, Simona Righi, Stefano A. Pileri

**Affiliations:** ^1^Hematopathology Section, Department of Hematology and Oncological Sciences “L. and A. Seràgnoli”, S. Orsola-Malpighi Hospital, University of Bologna, 40126 Bologna, Italy; ^2^Molecular Pathology Laboratory, Haematopathology Unit, Department of Haematology and Oncology “L. and A. Seràgnoli”, S. Orsola-Malpighi Hospital, University of Bologna, Via Massarenti, 9 - 40138 Bologna, Italy; ^3^Department of Human Pathology, University of Palermo, 90133 Palermo, Italy

## Abstract

Despite its well-known histological and clinical features, Hodgkin's lymphoma (HL) has recently been the object of intense research activity, leading to a better understanding of its phenotype, molecular characteristics, histogenesis, and possible mechanisms of lymphomagenesis. There is complete consensus on the B-cell derivation of the tumor in most cases, and on the relevance of Epstein-Barr virus infection and defective cytokinesis in at least a proportion of patients. The REAL/WHO classification recognizes a basic distinction between lymphocyte predominance HL (LP-HL) and classic HL (cHL), reflecting the differences in clinical presentation and behavior, morphology, phenotype, and molecular features. cHL has been classified into four subtypes: lymphocyte rich, nodular sclerosing, with mixed cellularity, and lymphocyte depleted. The borders between cHL and anaplastic large-cell lymphoma have become sharper, whereas those between LP-HL and T-cell-rich B-cell lymphoma remain ill defined. Treatments adjusted to the pathobiological characteristics of the tumor in at-risk patients have been proposed and are on the way to being applied.

## 1. Introduction

**Figure 1:**
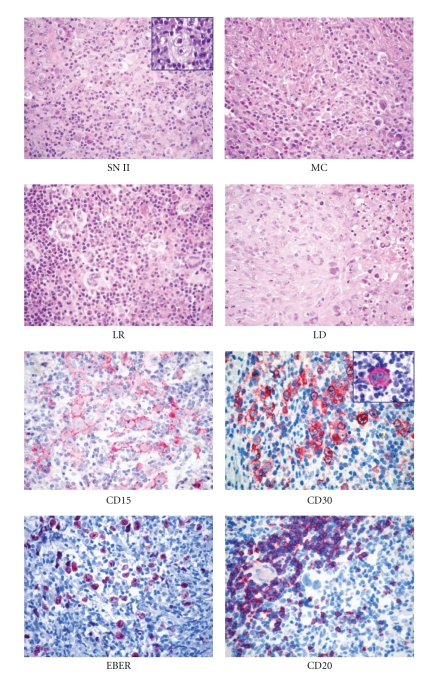
Histopathological features of classical Hodgkin lymphoma (cHL). At morphology (H&E staining), it is possible to distinguish nodular sclerosis (NS)*, mixed cellularity (MC), lymphocyte rich (LR), and lymphocyte-depleted (LD) subtypes. At immunophenotyping, cHL is typically CD15^+^, CD30^+∗^, possibly EBER^+^, and CD20^-/+^ (Olympus BX41 microscope, Olympus CAMEDIA C-7070 camera, magnification ×400, colours balanced after acquisition with Adobe Photoshop). *Note the typical Reed-Sternberg cells in the insets.

Hodgkin's lymphoma (HL) is a lymphoid tumor representing less than 1% of all *de novo* neoplasms occurring every year world wide [1]. Its diagnosis is based on the identification of characteristic multinucleated giant cells within an inflammatory milieu. These cells—termed Reed-Sternberg (RS) or diagnostic cells—represent the body of the tumor; they measure 20–60 *μ*m in diameter and display a large rim of cytoplasm and at least two nuclei with acidophilic or amphophilic nucleoli, covering more than 50% of the nuclear area (Figure 1). The tumoral population also includes a variable number of mononuclear elements—Hodgkin's cells (HCs)—showing similar cytological features to RS cells and neoplastic cell variants, each corresponding to a specific subtype of HL. Molecular studies have only recently shown that in most if not all cases RS cells, Hodgkin's cells, and cell variants belong to the same clonal population, which is derived from peripheral B cell [1–9]. Accordingly, the disease has been included among malignant lymphomas, and the term “Hodgkin's lymphoma” has been proposed [10–12]. 

However, although HRS cells are B lymphoid cells, they are unlike any normal B cell. Furthermore, their characteristics definitely contrast with a possible “stem cell” potential. Interestingly, it was recently shown that both the L428 and KM-H2 HL cell lines contained rare B-cell subpopulations responsible for the generation and maintenance of the predominant HRS cell population [13]. Intriguingly, the B cells within the HL cell lines expressed immunoglobulin light chain, the memory B-cell antigen CD27, and the stem cell marker aldehyde dehydrogenase (ALDH) [13]. Grippingly, CD27^+^/ALDH^++^ B-cells, clonally related to lymph node HRS cells, were also detected in the blood of HL patients. Indeed, though the significance of such circulating clonotypic B-cells has to be defined, the authors suggested that they may be the initiating cells for HL [13].

Although regarded as “diagnostic,” RS cells are not exclusive to HL because similar elements can be seen in reactive lesions (such as infectious mononucleosis), B and T cell lymphomas, carcinomas, melanomas, and sarcomas [14]. Thus, the presence of an appropriate cellular background—along with the results of immunophenotyping—is basic for the diagnosis. The reactive milieu, which can form up to 99% of the total population examined, consists of small lymphocytes, histiocytes, epithelioid histiocytes, neutrophils, eosinophils, plasma cells, and fibroblasts in different proportions, depending on the histological subtype of HL. It is sustained by an autocrine and/or paracrine production of several cytokines including, among others, IL-5, IL-8, IL-9, CCL-5, and CCL-28 (see below). The release of these molecules is also responsible for most of the symptoms recorded in patients with HL, in addition to the ability of the neoplastic cells to escape from growth controls and immunosurveillance. More recently, it has been proposed that hepatocyte growth factor and c-MET might constitute an additional signaling pathway between RS cells and the reactive cellular background, affecting adhesion, proliferation, and the survival of RS cells [15]. 

### 1.1. Histopathological Classification

In 1832, Sir Thomas Hodgkin provided the first macroscopic description of the process in a paper entitled “On some morbid appearances of the absorbent glands and spleen.” In 1898 and 1902, Carl Sternberg and Dorothy Reed independently described the typical “diagnostic” cells. In 1944, Jackson and Parker proposed the first comprehensive classification of the tumor (Table 1). However, this classification was subsequently found to be clinically irrelevant because most patients belonged to the granulomatous subtype and the response to treatment varied greatly from case to case.

In 1956, Smetana and Cohen identified a histopathological variant of granulomatous Hodgkin disease (HD), which had a better prognosis and was characterized by sclerotic changes; this variant was termed “nodular sclerosis HD” in the classification proposed by Lukes, Butler, and Hicks in 1964 (Table 1). This last classification, simplified at the Rye conference in 1965 (Table 1), has been used routinely over the past 35 years because of the high interpersonal and intrapersonal reproducibility and good clinicopathological correlations.

In 1994, in the light of morphological, phenotypic, genotypic, and clinical findings, HL was listed in the revised European-American lymphoma (REAL) classification [16] and subdivided into two main types: lymphocyte predominant (LP-HL) and common HL (CHL). CHL included the following subtypes: nodular sclerosis (NS-CHL), mixed cellularity (MC-CHL), lymphocyte depletion (LD-CHL), and the diffuse form of the lymphocyte rich CHL (LR-CHL) (Table 1). This approach has finally been adopted by the recently developed World Health Organisation (WHO) scheme (Table 1), which has promoted LR-cHL from a provisional entity to an accepted entity [1, 9, 17–20]. In this classification, the nodular form of LR-cHL has been included, as proposed by the European lymphoma task force [1, 9, 12, 17–22]. 

It is noteworthy that HL subtyping should be performed only in lymph node biopsies at the onset of the disease; in fact, chemotherapy and/or radiotherapy modify the histopathological picture by inducing a lymphocyte-depleted pattern.

## 2. Lymphocyte Predominant Hodgkin Lymphoma (LP-HL)

LP-HL represents 4%–5% of all HL cases [1, 9, 17–20] and differs greatly from the common type in terms of morphology, phenotype, genotype, and clinical behavior. The only feature shared by LP-HL and cHL is the low number of neoplastic cells. For a long time, after the adoption of the Lukes classification, the tumor was also called “nodular paragranuloma”, a designation coined by the Kiel group [23], based on the term “paragranuloma” introduced much earlier by Jackson and Parker. This designation intended to underline the differences existing between this type of HL and the remaining ones.

### 2.1. Clinical Findings

LP-HL displays features that are not generally encountered in cHL, which makes its clinical picture closer to that of “indolent” B-cell lymphoma [24]. First, it has a unimodal age distribution, with a single peak in the 4th decade, which contrasts with the two peaks of cHL, one in the 3rd and the other in the 7th decade [24]. The disease usually affects single cervical, axillary, or inguinal nodes rather than groups of nodes. Bone marrow involvement is found only occasionally during staging procedures in patients whose disease appears to be limited to a single node [24]; this pattern of spread differs from the orderly progression classically seen in cHL [25]. Involvement of the thymus is most unusual, unlike the other types of HL [24]. The tumor has a very indolent course, with prolonged disease-free intervals, despite a high rate of late relapses, which usually respond well to treatment [24, 26]. In addition, it can be associated with a diffuse large B-cell lymphoma (DLBCL), which has a more favourable outcome than de novo large B-cell lymphomas [24, 27].

### 2.2. Morphological Findings

**Figure 2:**
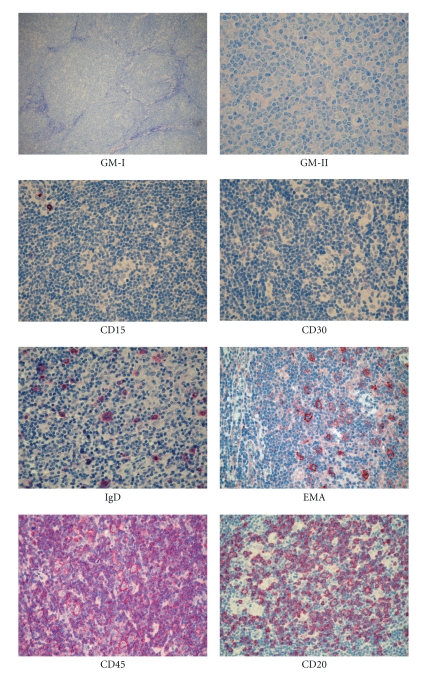
Histopathological features of lymphocyte predominant Hodgkin lymphoma (LP-HL). At morphology (GIEMSA staining), it is possible to appreciate the nodular morphology (GM-I) and the typical LP cells (GM-II, arrow). At immunophenotyping, it is typically CD15^−^, CD30^−^, EMA^+^, CD45^+^, CD20^+^, and possibly (10%–20% cases) IgD^+^ (Olympus BX41 microscope, Olympus CAMEDIA C-7070 camera, magnification ×400, colours balanced after acquisition with Adobe Photoshop).

In most instances, the growth is—at least in part—nodular (Figure 2), with the occurrence of a diffuse variant of the process being very rare [1, 9, 17–20, 24, 28]. The neoplastic population consists of large elements, called L&H (lymphocytic/histiocytic) or popcorn cells [24] (Figure 2). The former term has almost completely been abandoned in the light of the confirmed lymphoid derivation of the tumor and substituted by the LP (lymphocyte predominant) one in the IV edition of the WHO classification [1, 3–7, 9, 12, 17–20]. LP cells show nuclei resembling those of centroblasts, with a polylobular profile, finely dispersed chromatin, and small nucleoli, which are often adjacent to the nuclear membrane (Figure 2) [24]. Their cytoplasmic rim is narrow and basophilic when stained with Giemsa. Occasionally, neoplastic elements display the features of RS cells and/or of lacunar cells of NS-cHL and are associated with minimal sclerosis [24]; under these circumstances, immunophenotyping plays a fundamental role in the differential diagnosis between LP-HL and LR- cHL or NS- cHL. The reactive milieu consists of small lymphocytes with some plasma cells and epithelioid elements, which at times become so numerous as to mimic a histiocyte rich, large B-cell lymphoma (HCRBCL) [29]. According to the histological pattern, Fan et al. in 2003 proposed a subclassification of LP-HP into 6 categories (Table 2).

### 2.3. Progressively Transformed Germinal Centres

Progressively transformed germinal centres (PTGCs)—first described by Lennert in collaboration with Müller-Hermelink in 1978 [30]—are a peculiar form of follicular hyperplasia, which can be confused with LP-HL.

PTGCs occur in children and young adults, and these individuals reveal a slightly higher risk of developing LP-HL than the average population. PTGCs can precede, concur with, or follow LP-HL [23].

On morphological grounds, PTGCs are two to three times larger than reactive follicles and predominantly consist of small lymphocytes, mainly mantle cells, intermingled with some centroblasts and follicular dendritic cells (FDCs). PTGCs can be differentiated from LP-HLs because of the lack of LP elements and their cytological composition; they are composed of a mixture of B (CD20+) and T (CD3+) cells, histiocytes, and FDCs, which overall produce a “moth eaten” appearance [30, 31].

### 2.4. Phenotypic Findings

The neoplastic cells have a characteristic profile, which differs greatly from that of cHL [12, 16, 22]. In particular, they are CD45+, CD20+, CD22+, CD79a+, J chain+/−, epithelial membrane antigen (EMA)+/−, and CD15−. CD30 positivity is rare and, when detected, weak (Figure 2). Interestingly, a certain number of extrafollicular reactive blasts (smaller than the popcorn cells) are detected by the anti-CD30 antibodies; in the past, they have been misinterpreted as tumoral elements [22]. Popcorn cells regularly express OCT2 and BOB.1 [32]. The transcription factor Oct2 and its coactivator BOB.1 play a basic role in immunoglobulin synthesis by triggering the specific gene promoter [33] and are excellent tools for the identification of neoplastic cells in LP-HL, in addition to their differentiation from those of cHL, which are negative in almost all instances [32]. Although LP-HL is characterized by a more preserved B-cell phenotype compared to the classical variant, a certain degree of defectivity was described by Tedoldi et al. [34] that observed a downregulation of several markers associated with the B-cell lineage (CD19, CD37, CD79b, and LYN) and with the germinal center maturation stage (CD10, LCK, and PAG). 

**Figure 3:**
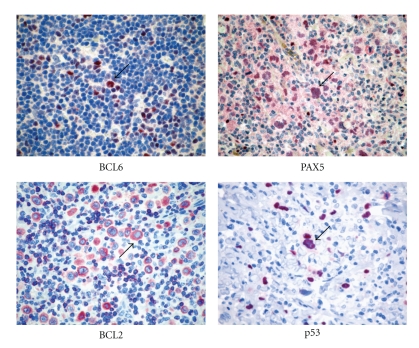
Immunophenotyping of Hodgkin lymphoma. Immunostains for BCL6, PAX5, BCL2, and p53 are shown. Please note positive staining in the diagnostic cells (arrows).

**Figure 4:**
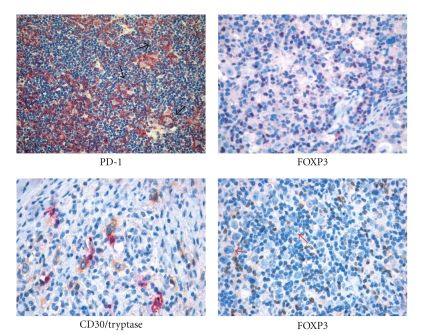
The reactive “milieau” in Hodgkin lymphoma. Mast cells and regulatory T cells populate the HL microenvironment showing spatial interaction with RS cells. Immunohistochemical staining for PD-1 and FOXP3 highlights the presence of several regulatory T cells intermingling with RS cells (arrows). Double immunohistochemistry for CD30 (yellow/brown) and mast cell tryptase (purple) shows the tight interaction of mast cells with RS cells.

The derivation of the tumor from germinal centres is supported by the following:

1. the expression of the *BCL6* gene product (Figure 3) [35], CD40, and CD86 by neoplastic cells [36, 37];
2. the occurrence of numerous CD4+/CD57+/PD1 T cells surrounding the popcorn cells, as seen in normal germinal centres and PTGCs (Figure 4) [37];
3. the presence of an FDC meshwork (CD21+/CD35+) within the nodules [38];
4. the global gene expression profile (see below) [39].

CD4+/CD57+/PD1 small lymphocytes resetting around typical CD20+/BCL6+ LP cells are indeed useful for the differential diagnosis with PTGC, LR- cHL, and TCRBCL (Figure 4). In addition, staining for LSP1, PU1, and IgD has to be considered. The latter, in particular, identifies a subgroup of cases (10%–20%) with peculiar epidemiological, phenotypical (IgD+, CD38+, CD27−, and IgM−), and clinical features [40, 41] (Figure 2).

### 2.5. Genetic Findings

Further evidence indicating that the tumor is derived from germinal centre B cells has been provided by recent molecular studies, based on the single cell polymerase chain reaction (PCR) [2–7, 12]. These studies have shown that LP cells in any given case represent monoclonal populations derived from germinal centre B cells, owing to the consistent occurrence of monoclonal *IGH* gene rearrangements and the high load of somatic mutations within variable region genes. Ongoing mutations are detected in about half of LP-HL cases; this finding—not observed in cHL—identifies mutating germinal centre cells as the precursors of the neoplastic elements [3, 6]. The pattern of mutation within these gene segments suggests that tumoral cells, their precursors, or both have been selected for expression of functional antigen receptors [3, 5, 6]. In addition, aberrant somatic hypermutation targeting PAX5, RHOH/TTF, PIM1, and MYC has been recorded in 80% of LP-HL cases, further supporting the GC derivation [42].

Recently, gene expression profile (GEP) analysis carried on isolated neoplastic cells indicated that LP cells possibly originate from germinal center B-cells at the transition to memory B cells [39]. In addition, LP cells showed a surprisingly high similarity to the tumor cells of TCRBCL and cHL, a partial loss of their B cell phenotype, and deregulation of many apoptosis regulators and putative oncogenes. Importantly, LP cells turned out to be characterized by constitutive NF*κ*B activity and aberrant extracellular signal-regulated kinase signalling [39].

Finally, to date, in situ hybridisation studies with Epstein-Barr virus (EBV) early RNA 1/2 (EBER1/2) probes, in addition to conventional Southern blot, PCR, and immunohistochemistry for the latent membrane protein 1 (LMP-1), have never detected EBV in the LP cells of LP-HL, in contrast to the neoplastic component of cHL [43, 44]. Isolated small lymphocytes from the reactive background carry EBV infection in 25% of cases of cHL [19].

## 3. Classic HL

This variant comprises about 95% of all HL cases and shows a typical bimodal age distribution, with a peak at 10–35 years of age and a second peak in late life [1, 9, 17–20]. It is characterized by a series of clinical, morphological, phenotypic, and genotypic features, which are integrated by specific findings in the four subtypes of the process (nodular sclerosis, mixed cellularity, lymphocyte depletion, and lymphocyte rich). 

### 3.1. Clinical Findings

cHL usually presents in the laterocervical lymph nodes, with peripheral extranodal involvement being very rare. About 50% of patients are in stage I or II. A mediastinal mass is seen in most patients with NS- cHL, at times showing the characteristics of “bulky” disease. Systemic symptoms—fever, night sweats, and body weight loss—are detected in approximately 25% of patients. In contrast to earlier reports, the histological subtype is not regarded as a major prognostic indicator. Without treatment, cHL has a moderately aggressive clinical course. With the present treatments, 70%–80% of cases show long-term survival [45–49].

### 3.2. Morphological Findings

In cHL, typical Hodgkin's and Reed-Sternberg (H&RS) cells (Figure 1) can be easily detected; their number (from few to many) differs from case to case. They may be associated with peculiar cell variants and are found within an inflammatory milieu, related to the histological subtype (see below). The lymph node structure is largely effaced although remnants of normal follicles can be detected in some cases. The type of structural alteration is indeed characteristic in NS-cHL.

### 3.3. Phenotypic Findings

In 1982, Schwab et al. described a new monoclonal antibody, termed Ki-1, whose reactivity seemed restricted to H&RS cells and a small subset of normal lymphocytes with perifollicular location. However, the extensive application of the antibody showed that it was not specific to H&RS cells, as originally thought, but reacted with a variety of lymphoid tumors, including anaplastic large-cell lymphoma (ALCL) [50–52] and nonlymphoid tumors such as embryonic carcinoma, pancreatic carcinoma, nasopharyngeal undifferentiated carcinoma, and malignant melanoma [14, 53, 54]. Therefore, the immunophenotypic diagnosis of HL should always be based on the application of a panel of antibodies, including reagents against cytokeratins, melanoma-associated antigens, carcinoembryonic antigen, and placental alkaline phosphatise [14].

Expression of the CD30 molecule by H&RS cells is seen in more than 98% of cHLs (Figure 1) although the intensity of the immunostaining can vary from one case to another, and even within the same case. Interestingly, the antigen is masked by fixation (especially prolonged fixation in formalin or fixation in B5); thus, very efficient antigen retrieval techniques are required to achieve reliable results in routine material [55] 

Notably, the CD30 molecule has also been proposed as a possible target for specific antibodies conjugated with plant toxins and administered to patients with cHL for therapeutic purposes; preliminary studies have shown these immunotoxins to have remarkable cytotoxic activity [56–58]. 

CD15 is another valuable marker for H&RS cells (Figure 1) and is detected in about 80% of patients with cHL [51, 59]. CD15 is characteristic, but not specific, for H&RS cells because it can be detected (although rarely) in B and T cell lymphomas and in nonlymphoid tumors [51, 59, 60].

H&RS cells usually lack CD45 and EMA expression [61–64], whereas B and T cell markers are seen in a proportion of cases. In particular, CD20 (Figure 1) is found in 30%–40% of cHL cases (usually EBV negative) [63], and CD79a is found even less often [65–67]. Positivity (usually weak) for one or more T cell marker is detected in a minority of H&RS cells in some cases [68, 69]. Under these circumstances, single cell PCR studies have so far shown T-cell receptor (TCR) gene rearrangement in only three instances, with clonal Ig gene rearrangements occurring in most cHL cases with T-cell marker expression [8, 70]. In contrast to that seen in LP-HL, the elements of cHL show variable expression of the BCL6 molecule [17]. In addition, they are usually positive for PAX5/BSAP and IRF4 and negative for BOB.1 and OCT2 [7, 8, 71].

Antibodies against the nuclear-associated antigens Ki-67 and proliferating cell nuclear antigen (PCNA) stain most H&RS cells, suggesting that a large number of neoplastic cells enter the cell cycle [72, 73]. However, in spite of this, tumor cells do not rapidly overwhelm the reactive component [72, 73]. This phenomenon has found a satisfactory explanation in the studies of Leoncini and coworkers, who have shown that H&RS cells have a defect in cytokinesis [74]. In fact, only a minority of the cycling elements undergo effective mitosis, and a proportion of the cells that do not enter into the cell cycle undergo apoptosis, a step partly regulated by the BCL2 and p53 gene products [74]. 

On prognostic grounds, it has also been proposed that chemoresistance and the tendency to relapse are influenced by the expression of BCL2, p53, p21, and PCNA [75, 76] (Figure 3). In general, tumors with H&RS cells showing expression or overexpression of one or more of these molecules seem to have a poor response to the treatment and/or short survival time.

### 3.4. The Babel of HL Microenvironment

In classical Hodgkin's lymphoma, the tumor-associated microenvironment endows a central role, as far as the pathobiology, diagnosis, prognosis, and even therapy are concerned. In most HL cases, nonneoplastic cellular components, comprising immune and stromal cells, account for the vast majority of the tumor burden (usually more than 90%). In such a setting, in which neoplastic cells are dispersed among reactive elements, the “pressure” of the microenvironment over the neoplastic clone may be perceived on mere morphology (Figure 1). Indeed, a strong reciprocal influence exists between RS cells and the diverse types of reactive cells of the microenvironment.

The composition of the cellular milieu associated with HL includes cells of the innate and adaptive branches of the immune system, such as granulocytes, macrophages, mast cells, T and B lymphocytes, plasma cells, and mesenchymal elements, namely endothelial cells, adventitial reticular cells, fibroblasts, and follicular dendritic cells forming an intricate vascular and perivascular meshwork.

The composition and amount of reactive components may vary significantly among the diverse histological subtypes of classical HL and even at discrete stages of the disease course. For example, the reactive background is most pleomorphous in HL cases of the mixed cellularity histotype, where inflammatory elements efface the lymph node architecture, while it is mainly composed of lymphocytes organized within preserved or regressed lymphoid follicles in cases of the lymphocyte-rich type [1, 9, 17–20]. In these two conditions, profoundly different interactions between neoplastic cells and the surrounding reactive microenvironment can be predicted. In the nodular sclerosis variant, the presence of a prominent mixed inflammatory background may be progressively reduced by the accumulation of collagen fibrosis suggesting a dynamic process of tissue remodelling [1, 9, 17–20].

H&RS cells have a major role in the orchestration of the microenvironment milieu associated with HL. They can directly induce the recruitment of several immune cell types from the peripheral circulation and also trigger the local expansion of diverse cellular subsets. Specifically, RS cells synthesize a whole plethora of soluble mediators with chemotactic activity such as the cytokines and chemokines IL-5, IL-8, IL-9, CCL-5, and CCL-28 involved in the recruitment of granulocytes, mast cells and macrophages, and IL-7, CCL-5, CCL-17, CCL-20, and CCL-22, effectors of lymphocyte recruitment and expansion [77, 78]. Recruitment of infiltrating immune cells is also boosted by reactive cells themselves and particularly by macrophages and mast cells synthesizing CCL-3, CCL-4, and CCL-8 chemokines [78, 79]. RS cells and recruited or locally-expanded immune and stromal cells give rise to a complex and dense network of signals mediated by direct cell contact and soluble mediator synthesis. Most of these signals have their relevant final effect in the delivery of a prosurvival feedback to RS cells. These include the engagement of surface CD40 and CD30 molecules expressed on RS cells by CD40L-expressing T lymphocytes, NK cells, and mast cells, which are often found surrounding RS cells, and by CD30L expressed on B lymphocytes, granulocytes, macrophages, and mast cells [80, 81]. 

Besides directly interacting with neighbouring cells through these pivotal axes, RS cells are able to sense growth and survival signals coming from the growth factor milieu, owing to the expression of a broad range of receptors including IL-7R, IL-9R, IL-13R, TACI, and CCR5 [78]. The combination of paracrine and autocrine mechanisms grants a high degree of redundancy to RS cell stimulation, thus making any attempt of therapeutic interference with these mechanisms challenging. Along with growth factors, also proinflammatory cytokines and mediators can sustain RS cell expansion through the activation of pathways converging into the NFkB hub, such as those triggered by IL-6R, TACI, RANK, TNFR-1, Cys-LT receptors, and NOTCH-1 engagement [82]. These proinflammatory spurs may be either derived from the microenvironment (e.g., leukotriene production by mast cells and NOTCH-1 ligand expression by stromal cells) or originate from both RS cells and reactive elements (e.g., IL-6, TNF). 

If RS cells are the main beneficiaries of this Babel of cellular and molecular interactions, they are not the sole targets. Indeed, the stromal components of the microenvironment may be induced towards significant changes characterized by the proliferation of follicular dendritic cells (FDC) and fibroblasts and microvascular sprouting [1, 9, 17–20]. Stromal changes can be directly supported by RS cells through the synthesis of cytokines active on the stroma, such as IL-6, IL-7, IL-8, IL-13, and TNF. Nevertheless, in the stromal remodelling of HL-infiltrated tissues, a major role is played also by cells other than RS ones, mostly macrophages and mast cells that are particularly active in regulating angiogenesis and extracellular matrix (ECM) deposition. Moreover, stromal FDCs, which are a relevant source of stimulatory signals for the HL neopastic clone of GC derivation, as well as for reactive T and B cells, are mainly expanded through the synthesis of CXCL-13 and IL-21 by follicular helper T cells expressing PD-1 and CXCR-5 [83].

**Figure 5:**
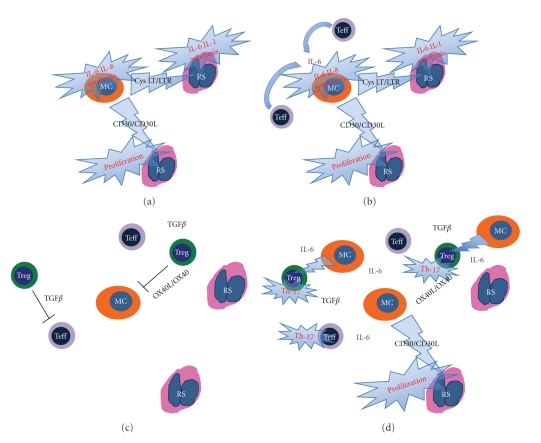
Schematic representation of the reciprocal contribution of mast cells (MC), effector T cells (Teff), and regulatory T cells (Treg) to the HL-associated immunological microenvironment. (a) Mast cells, as other innate immune players directly sustain RS cell proliferation and proinflammatory mediator synthesis. (b) This effect is boosted by Teff that contributes to mast cell activation and amplifies the inflammatory spur. (c) Treg can interfere with the activation status of both Teff and MC through the OX40/OX40L axis and TGF-*β* release eventually limiting the delivery of the proliferation/survival signal to RS cells. (d) However, when the microenvironment is diverted towards marked inflammation owing to the abundant presence of activated MC, the regulatory function of Treg may prove inadequate to restore the balance between pro- and anti-inflammatory stimuli, and Treg can even boost inflammation through TGF-*β* release and Th17 generation.

The influence of the cellular components of the microenvironment and that of the elaborate network of interactions they produce, on the clinical course of HL, has progressively emerged over the past decades. In particular, the detrimental contribution of mast cells and CD68+ macrophages to HL patients' survival has been clearly established [84, 85]. Though the precise mechanisms through which these cell types favour neoplastic clone progression are not entirely characterized, they are likely linked to the ability of mast cells and macrophages to induce and maintain a proinflammatory microenvironment. In this light, we could hypothesize that the contribution of Tregs to HL might be function of the microenvironment polarization. Indeed, Tregs may limit the inflammatory spur of other cells of the immune system (including T effectors) by releasing IL-10 and TGF-b, and this beneficial effect may prevail over the impairment of an effective T-cell-mediated response, as far as the outcome of HL is concerned (Figure 5). Nevertheless, when the HL-associated environment is diverted towards marked inflammation owing to the abundant presence of mast cells and macrophages, the regulatory function of Tregs may prove inadequate to restore the balance between pro- and anti-inflammatory stimuli, and Tregs can even boost inflammation through TGF-b release and Th17 generation (Figure 3). Under these circumstances, a direct role for mast cells in the Treg contrasuppression and Th17 deflection can be envisaged as both mast cells and Tregs populate HL-infiltrated areas, and their interaction is therefore possible (Figure 4).

### 3.5. Genetic Findings

The origin of the RS cells of HL has long been a mystery [86]. As previously discussed in the LP-HL section, micromanipulation of single RS cells from tissue sections and PCR analysis of the cells for rearranged *IGH* genes have shown that most of both LP-HL and cHL cases represent clonal populations of B-cell lineage [2–7, 12]. In contrast to that seen in LP-HL, ongoing mutations of *IGH* genes are not detected in cHL [7]. On the other hand, the presence of aberrant somatic hypermutation (ASH) targeting PAX5, RHOH/TTF, PIM1, or MYC in 50% of cases further supported the origin from GC cells [42]. Based on the results obtained in a small series of cases, emphasis was instead given to the occurrence of mutations resulting in stop codons within originally functional variable region gene rearrangements [6]. Such mutations are expected to occur in variable region genes of germinal centre B cells, but under physiological conditions, “crippled” germinal centre cells (incapable of functional antibody expression) rapidly undergo apoptosis. RS cells might also have other mutations that can be crippling but may not be easy to find (e.g., replacement mutations interfering with antigen binding or heavy, and light-chain pairing) [6]. However, by analyzing a large number of cases, Marafioti et al. found that crippling mutations are absent from 75% of cHL s, indicating that crippling mutations cannot be responsible for the general absence of the *IGH* transcripts [7], which might be the result of downregulation of the synthesis of the transcription factors BOB.1 and OCT2 (see above) [7, 8]. 

Recently, some studies have pointed to the possibility that the nuclear transcription factor NF*κ*B is involved in the protection of H&RS cells from apoptosis, which would be expected because of their inability to produce immunoglobulins [87]. The persistent activation of NF*κ*B in H&RS cells might be caused by defects in members of the I*κ*B family, which are the natural inhibitors of NF*κ*B [87–90], or by the aberrant activation of I*κ*B kinase [91]. To this regard, interestingly, it was recently shown that the tumor suppressor gene *TNFAIP3*, encoding for A20, a negative regulator of NF*κ*B, is frequently mutated in cHL, leading to NF*κ*B constitutive activation [92]. Conversely, it appeared to be not affected in LP-HL [93].

 In contrast, despite the frequent expression/overexpression of p53 by neoplastic cells, no mutations of exons 4–8 of the *TP53* gene have been detected in H&RS cells [94].

No specific cytogenetic abnormalities have been reported in cHL because aberrations vary from one case to another, with frequent intraclonal variability, thus suggesting chromosomal instability [95]. Some tumors show 14q alterations, as seen in B-cell lymphomas, but without the occurrence of the t(14;18) translocation [95]. On the other hand, recent studies, by using high-throughput techniques, identified recurrent imbalances with correlations to treatment outcome [96]. In particular, genomic loci containing genes encoding for NFkB molecules as well as proteins involved in drug resistance (i.e., ABCC1) were found to be frequently altered in microdissected H&RS cells, such abnormalities correlating with the overall survival [96]. Interestingly, a recent study documented the association between 9p24.1 copy number and PD-1 ligand expression in cHL and the frequent involvement of the Janus kinase 2 (JAK2) locus. Thus, the PD-1 and JAK2 pathways were defined as potential novel therapeutic targets [97].

In addition, recently, by using comparative genomic hybridization arrays (aCGH) on HL cell lines, a complex patterns of rearrangements was demonstrated including 35 previously uncovered aberrations [98]. Among others, the homozygous deletion of 15q26.2, amplification of the STAT6 gene, and a 2.35 Mb deletion at 16q12.1, putatively defining a small critical region for the recurrent 16q deletion in cHL, appeared to be of interest. Notably, the latter region contains the CYLD gene, a known suppressor gene of the NFkB pathway [98].

Subsequently, an aCGH study applied to primary cHL samples identified the recurrent involvement of several regions by either amplification (2p12-16, 5q15-23, 6p22, 8q13, 8q24, 9p21-24, 9q34, 12q13-14, 17q12, 19p13, 19q13, and 20q11) or losses (Xp21, 6q23-24, and 13q22) [99]. Notably, several gained regions included genes known to be constitutively expressed in cHL. Among these, gains of STAT6 (12q13), NOTCH1 (9q34), and JUNB (19p13) were confirmed [99].

EBV studies reveal viral integration in the genome of cHL tumor cells in a variable proportion of patients (20%–80%), depending on the histotype. In particular, in Western countries, 20%–40% of NS and LD cases and 50%–75% of MC cases show expression of LMP-1 and/or EBER1/2, (Figure 1) but not EBV- encoded nuclear antigen 2, thus showing a pattern characteristic of latency type II EBV infection [100, 101]. Interestingly, these figures can vary greatly according to the geographical area examined, as recently shown by Leoncini and coworkers, who found significant differences in the incidence of EBV between patients with cHL from Kenya and Italy (92% versus 48%) matched for age and histotype [102]. The type of EBV strain also varies between different geographical areas; in developed countries strain 1 prevails, whereas strain 2 is most prevalent in developing countries [103]. HLs that are positive for EBV at diagnosis are usually also positive at relapse, with persistence of the same EBV strain [104]. The exact role of EBV in the pathogenesis of cHL (transforming agent? cofactor for the maintenance of malignant growth?) is still open to question [44].

Recently, a couple of gene expression profile (GEP) studies dealt with HL, by focusing on the identification of novel prognostic features. First, Sanchez-Aguilera et al. [105] have identified a gene signature able to distinguish patients with classical Hodgkin lymphoma with good and poor prognosis; it consists of gene expressed by neoplastic cells related to growth/apoptosis and genes of tumor microenvironment involved mainly in immune response and matrix remodelling. The GEP results has been subsequently validated by immunohistochemistry in and independent series of samples, using antibody directed against products of selected representative genes (SAP, STAT1, RRM2, CDC2, MAD2L1, ALDH1A1, Top2*α*, and PCNA). This suggests that both the biology of neoplastic cells and the characteristic of the background could be determining factors for the clinical behaviour of the neoplasia and the success of therapy. The possible identification in the near future of novel biological markers able to identify patients with different prognosis would be a dramatic improvement in HL patients management. In fact, at present, the best prognostic indicator is probably represented by PET-scan evaluation after two course of chemotherapy [106]. As a matter of fact, patients are identified as high risk, requiring different therapeutic strategies only after having received a significant (and useless) toxic load.

More recently, Steidl et al. studied a large series of HL and identified tumor microenvironment as a major determinant of clinical outcome [85]. In particular, a molecular signature representative of macrophage infiltration was significantly associated with overall survival [85]. Importantly, such finding was confirmed in an independent cohort of patients by studying the tumor-associated macrophages by immunohistochemistry (CD68 staining) [85].

### 3.6. Nodular Sclerosis

#### 3.6.1. Morphological Findings

NS is the most frequent subtype of cHL in Italy and the USA, where it corresponds to 75%–80% of all HL cases; however, the incidence of these subtypes varies greatly among other geographical areas [107–109]. As stated by Lukes et al. in 1966, the tumor is characterized by sclerosis, lacunar cells, and nodular pattern.

**Sclerosis**

Fibrotic phenomena always occur in NS- cHL; they more often correspond to the formation of broad collagen bands, which originate from a regularly thickened lymph node capsule (Figure 1) and subdivide the lymphoid parenchyma into large nodules, at times visible at gross examination. Fibrotic tissue displays a typical birefractive green colour at polarised light microscopy, a finding never seen in LD-cHL.

**Lacunar Cells**

These cells are characteristic of NS-HL. Lukes et al. originally described them as large elements with polylobular nuclei, small-to-medium-sized nucleoli, and a wide rim of clear or slightly acidophilic cytoplasm, which is very sensitive to formalin fixation. This last factor causes perinuclear condensation of the cytoplasm, which remains connected to the cell membrane via some narrow filaments, forming empty “lacunar” cytoplasmic spaces. In fact, lacunar cells display a much higher degree of pleomorphism than was originally thought; they may be unilobular, multilobular, and/or showing huge nucleoli, which are indeed similar to those of typical RS cells. This morphological variability seems to depend on the characteristics of the inflammatory component present in each case [110]. Although lacunar cells are easily detected, H&RS cells are rare and their identification may involve a long search. Finally, it should be stressed that some neoplastic elements appear to be “mummified” because of apoptotic changes.

**Nodular Pattern**

The nodules, which should be detected in at least part of the lymph node involved, can contain foci of necrosis and can be very variable in terms of inflammatory cell component (from lymphocyte predominance to lymphocyte depletion).

**NS-CHL: Cellular Phase**

In NS- cHL, the amount of collagen fibres varies greatly from one case to another. In the so-called cellular phase, there is a clear cut tendency to nodule formation without overt collagen band deposition. However, there are typical lacunar cells, often located at the periphery of the nodules or around residual follicles. The reactive component mainly consists of small lymphocytes bearing the phenotype of mantle B cells (CD20+, CD79a+, CD5+, IgM+, IgD+, and CD3−) [108, 111]. The secretion of cytokines by neoplastic cells is currently believed to cause the progressive attraction of T cells, histiocytes, plasma cells, and eosinophils, which give rise to nodules replacing the pre-existing follicles and produce the typical pattern of NS-cHL. Within the nodules, there are numerous FDCs, which seem to represent a favourable prognostic indicator [112, 113].

**NS-CHL Syncytial**

The term “syncytial” NS- cHL was coined by Butler in 1983 and then reproposed by Strickler et al. in 1986 [114]. This variant is thought to form 16% of all NS-cHL cases [115] and to run a more aggressive clinical course [109, 115], as suggested by the occurrence of mediastinal bulky disease and stage III/IV in 88% of the patients. At light microscopy, it is characterized by large sheets of neoplastic cells (partly with a lacunar appearance), which may undergo central necrosis [114]. The differential diagnosis (which includes non-Hodgkin's lymphoma, metastatic melanoma, carcinoma or sarcoma, thymic carcinoma, or germ cell tumors) requires the application of an adequate panel of antibodies, which allows the identification of the characteristic phenotype of the tumoral cells: CD3−, CD15+, CD20−/+, CD30+, CD45−, CD79a−, cytokeratin negative, PLAP−, protein S-100−, HMB.45 melanoma-associated antigen negative, EMA−, and ALK−.

**Histological Grading of NS-HL**

The British national lymphoma investigation (BNLI) group has repeatedly proposed that NS-cHL should be subclassified into two grades: grade II tumors seem to represent 15%–25% of all NS-cHL cases and to run a more aggressive clinical course [111, 116, 117], a finding not confirmed by all studies [112, 118, 119]. In the recently developed WHO scheme, the BNLI grading system has been maintained to test its real prognostic value on larger series [1, 9, 17–20]. It is based on the degree of cellularity of the nodules, the amount of sclerosis, and the number and atypia of neoplastic cells. The term grade II is applied to cases showing one of the three following patterns:

1. more than 25% of the nodules have a cellular composition consistent with the pleomorphic or reticular subtype of NS-cHL /LDV;
2. more than 80% of the nodules show a fibrotic or fibrohistiocytic composition;
3. more than 25% of the nodules contain numerous large bizarre or anaplastic cells, in the absence of depletion of the reactive small lymphoid component.

### 3.7. Mixed Cellularity

This histotype was originally described by Lukes et al. as intermediate between LR and LD- cHL. Later, Lukes included in this category all the cases that according to his criteria remained unclassified, transforming it into a “basket”.

About 15%–25% of cHL cases belong to this category. The histological picture is characterized by diffuse growth with a frequent paracortical location. The capsule is not often involved and necrosis seldom occurs. The term MC-cHL reflects the cellular composition of the reactive milieu, which consists of plasma cells, epithelioid histiocytes, eosinophils, and T cells (CD3+, CD57−), which form rosettes around neoplastic elements (Figure 4). The tumor cells correspond to H&RS cells and are numerous and easy to find, without lacunar or popcorn variants. Some neoplastic elements, as in the NS subtype, appear to be “mummified” because of apoptotic changes.

#### 3.7.1. Morphological Variants of MC

**Interfollicular Variant**

This variant is rarely seen and probably represents partial lymph node involvement by HL. It is characterized by the occurrence of numerous H&RS cells around reactive follicles, which display germinal centres either in the second phase of development [120] or in regressive transformation. These germinal centres usually resemble those seen in hyaline-vascular Castleman's disease and are probably related to the release of cytokines, such as IL-6 [121], by H&RS cells [122]. This unusual variant of MC-HL should be taken into consideration to avoid possible confusion with follicular hyperplasia or Castleman's disease [120, 123].

**Epithelioid Cell Rich Variant**

This variant is relatively common and shows a prominent epithelioid cell reaction with granulomata formation and occasional Langhans cells. In this context, typical H&RS cells are always detected, at times after a laborious search. It should be differentiated from the so-called Lennert's lymphoma because of the dramatic differences in terms of treatment between the two entities [111, 124].

### 3.8. Lymphocyte Depletion

This variant is very rare, accounting for about 1% of HL cases, and shows the worst clinical behavior and prognosis. In most instances, it presents in stage III-IV. B-cell symptoms and bone marrow involvement are detected in 50% of cases [125, 126]. At microscopic examination, it is characterized by paucity of the lymphoid component, absolute or relative abundance of RS cells, and variable fibrotic reaction. According to Lukes and Butler, two subtypes of LD-HL can be distinguished: fibrotic and reticular/sarcomatous.

**Fibrotic Variant**

This results in the complete effacement of the nodal structure with possible capsule preservation. At microscopic examination (Figure 1), it shows the following distinctive features: (1) low cellular density with scarce, although variable, amounts of small lymphocytes; (2) prominent diffuse reticulin fibre formation without organised birefringent collagen bands [123], which tend to include single neoplastic elements and are associated with the deposition of amorphous material (precollagen) around sinusoids; (3) a high variability in the number of H&RS cells, the detection of which sometimes requires a long and labourious search.

At low power, the histopathological picture can resemble the depletion phase of HIV lymphadenopathy [127]; therefore, careful node examination is needed to make a firm diagnosis [128].

**Reticular or Sarcomatous Variant**

This is characterized by extremely large numbers of H&RS cells, some of which appear to be “mummified”. The growth results in diffuse effacement of the normal lymph node structure; small lymphocytes, plasma cells, histiocytes, and granulocytes are scanty; foci of necrosis are usually encountered, although their extent varies from one case to another.

### 3.9. Lymphocyte Rich Classic Hodgkin's Lymphoma

Several reports have underlined the existence of HL cases with a lymphocyte predominant background, but differing from the prototypic description of LP-HL because of the presence of some eosinophils, sclerosis, typical H&RS cells, or aberrant phenotypic features, such as the expression of CD30 and CD15 [23, 38, 103, 107, 129]. In 1994, the ILSG included in the REAL classification a provisional entity called “lymphocyte rich common Hodgkin's disease”, which was thought to have a diffuse growth pattern in most instances (Table 1) [16]. Following two workshops held by the European Association for Haematopathology in 1994 and the European lymphoma task force in 1995, the existence of LR- cHL has been accepted and expanded by the recognition of two subtypes of the tumor, nodular and diffuse, which should be differentiated from LP-HL and TCRBCL (Table 1) [1, 17–20, 22, 130–133].

On morphological grounds [19], most LR- cHL cases are characterized by a nodular background, with admixed histiocytes and absent neutrophils and eosinophils closely resembling nodular LP-HL, particularly at low power. Furthermore, a varying proportion of the neoplastic cells can exhibit features of popcorn elements. However, in contrast to LP-HL, many lymphomatous cells have the cytomorphological features of classic H&RS cells, and the nodular structures frequently show small germinal centres at their periphery. Focal areas of sclerosis can sometimes be seen.

At phenotypic analysis [19], the neoplastic cells usually express CD30 and CD15. CD20 and CD79a positivity is found in 32.5% and 8.7% of cases, respectively—figures that are much lower than those observed in LP-HL. In addition, there is a complete absence of J chain in all instances and a weak expression of EMA in only a few cases. About 50% of the examples of LR- cHL harbour EBV positive H&RS cells. The reactive component consists of abundant mantle B cells, with surface IgD and IgM expression, and variable amounts of CD3+ T cells, which produce rosettes around neoplastic elements, but they seldom express CD57. CD21 immunostaining reveals a loose, ill defined meshwork of FDCs, which becomes much denser and sharper around the small residual germinal centres, when present.

On clinical ground, patients with LR- cHL differ from those with NS- cHL or MC- cHL; they are usually older than 50 and display a higher incidence of stages I–II and a subdiafragmatic location. In contrast, they rarely have bulky disease, B-cell symptoms, or mediastinal or extranodal involvement [19, 134]. Thus, the clinical profile of LR- cHL is closer to that of LP-HL although it has a lower frequency of stages I-II and splenic infiltration is more common. When compared with other types of cHL, LR-cHL gives rise to more frequent late relapses although these do not behave aggressively.

Owing to its peculiar clinicopathological features, LR-cHL has been quoted as an accepted entity in the recently developed WHO scheme [1, 9, 17–20].

### 3.10. Unclassifiable HL

In cases with lymph node partial involvement, small amounts of tissue available, or extranodal location, the classification of HL can be difficult or even impossible. In the past, these problematical cases were usually included in the MC subtype. Because it is useful to keep the subtypes of HL as homogeneous as possible for prospective clinicopathological studies, both the REAL classification and the WHO scheme list cases with ambiguous features or insufficient bioptic material as HL unclassified.

### 3.11. Lymphomas with Intermediate Features between cHL and DLBCL

This new distinct entity (previously called grey zone lymphoma) has been introduced in the 4th edition of WHO classification of Tumours of Haematopoietic and Lymphoid Tissues and includes B lineage lymphoma with overlapping clinical, morphological, and immunophenotypic features between cHL and DLBCL, in particular primary mediastinal large B-cell lymphoma (PMBL) [135]. The clinical onset is often represented by a large anterior mediastinal mass which may involve the lung and may be associated to a vena cava syndrome. The disease is frequently more aggressive than cHL and PMBL. Morphologically the neoplastic population is pleomorphic with a sheet-like growth pattern; areas richer in lacunar and Hodgkin cells and others more similar to a DLBCL are frequently observed. Similarly to DLBCL, the immunophenotype is characterized by the expression of CD45, of B-cell antigens CD20 and CD79a, and transcription factors PAX5, BOB1, and OCT2 but is also associated to cHL markers CD30 and CD15 positivity [135]. Sometimes a neoplasia resembling morphologically a PMBL but negative for CD20 can occur; in these instances, a diagnosis of lymphoma with intermediate features between cHL and DLBCL can be made if supported by CD15 and/or EBV positivity [135]. 

Of note, some of EBV-positive DLBCL of the elderly are pathologically similar to HL and should be considered in the differential diagnosis of EBV-positive HL [136, 137].

## 4. Extranodal Involvement by HL

Although the onset is typically nodal, HL can secondarily affect extranodal organs and tissues. The criteria for the diagnosis of HL at extranodal sites vary greatly depending on the clinical history and the type of tissue involved. In fact, in needle biopsies taken from the bone marrow and liver during staging procedures, the diagnosis of HL can confidently be made according to “minimal criteria”; that is, by the detection of HC in the appropriate cellular milieu [127]. In contrast, the diagnosis of HL at other extranodal sites needs the recognition of typical “diagnostic” cells and appropriate phenotypic markers, especially in patients with no previous history of HL.

**Expert Opinion**

Thanks to the results provided by immunophenotypic and molecular studies, Hodgkin's lymphoma (HL) is now basically considered to be a germinal centre-related B-cell lymphoma. Notably, significant differences exist between LP-HL and cHL (which includes the NS, MC, LR, and LD subtypes) in terms of natural history, the relation to Epstein-Barr virus, cell morphology, phenotype, molecular characteristics, and clinical behavior. Further, although the borders between HL and ALCL have recently become sharp, the differential diagnosis between LP-HL and T-cell rich B-cell lymphoma remains at times problematical. Interestingly, the lack of immunoglobulin (Ig) production, which is characteristic of cHL, is more often the result of defective expression of transcription factors, such as OCT-2, BOB.1, and PU.1, although at times it is caused by the occurrence of crippling *IGH* gene mutations. Finally, the search for morphological, phenotypic, and/or kinetic factors that may herald a poor response to conventional treatments is felt necessary, aiming to design and to apply more effective ad hoc strategies in selected cases. In particular, strategies aimed at interfering with the crosstalk between RS cells and their cellular accomplices should take into account that the same cellular subset may play different, even opposite, functions according to the signals it senses. This suggests a note of caution regarding the adoption of therapies able to alter the composition of the HL-associated microenvironment, such as monoclonal-antibody-based humoral immunotherapies (e.g., Rituximab) or immunomodulatory drugs (e.g., lenalidomide) [138, 139]. On the other hand, other target therapies such as anti-CD30 antibodies and anti-NFkB drugs may provide a significant benefit in the limited but still significant fraction of chemoresistant HL patients.

##  Funding

The authors have no conflictng financial interests to declare.

## Figures and Tables

**Table 1:** Hodgkin's lymphoma (HL) classification schemes.

*Jackson and Parker classification *	
Paragranuloma	
Granuloma	
Sarcoma	

*Lukes and Butler classification*	
Lymphocytic and/or histiocytic, nodular	
Lymphocytic and/or histiocytic, diffuse	
Nodular sclerosis	
Mixed cellularity	
Diffuse fibrosis	
Reticular	

*Rye conference classification*	
Lymphocyte predominance	
Nodular sclerosis	
Mixed cellularity	
Lymphocytic depletion	

*Revised European-American lymphoma (REAL) classification*	
Nodular lymphocyte predominance nodular/diffuse	
Classic HL	
Nodular sclerosis	
Mixed cellularity	
Lymphocyte depletion	
Lymphocyte rich classic HL diffuse (provisional entity)	

*World Health Organisation scheme*	
Nodular lymphocyte predominant HL	
Classic HL	
Nodular sclerosis HL (grades 1 and 2)	
Lymphocyte-rich classic HL*	
Mixed cellularity HL	
Lymphocyte-depleted HL	
Unclassifiable classic HL	

*This includes a nodular (common) and a diffuse (rare) form in contrast to the REAL classification.

**Table 2:** Classification of LP-HL.

Classical nodular pattern, B-cell rich	
Serpiginous/interconnected nodular pattern	
Nodular, with prominent extra nodular B cells	
Nodular, with T-cell rich background	
Diffuse pattern (TCRBCL-like)	
Diffuse, “moth eaten” with B-cell-rich background	

According to Fan et al., (Am J Surg Pathol 2003).
